# Physical activity and nicotine dependence among female college smokers: a serial mediation model of smoking refusal self-efficacy and smokers’ identity identification

**DOI:** 10.3389/fpsyg.2026.1914403

**Published:** 2026-08-05

**Authors:** Xiaojun Song, Xiangzheng Meng

**Affiliations:** 1 School of General Education, Liaoning University of International Business and Economics, Dalian, China; 2 School of Physical Education, Liaoning Normal University, Dalian, China

**Keywords:** nicotine dependence level, physical activity, serial mediation, smokers’ identity identification, smoking refusal self-efficacy

## Abstract

**Introduction:**

This study aimed to investigate the association between physical activity and nicotine dependence level among female college smokers, and to examine the Serial Mediation Model of smoking refusal self-efficacy and smokers’ identity identification.

**Methods:**

A cross-sectional survey was conducted among 673 female college students who were current smokers (defined as smoking at least one cigarette per day in the past 30 days), recruited from 30 universities across various regions of China using stratified random sampling. Participants completed the Physical Activity Rating Scale, the Fagerström Test for Nicotine Dependence, the Smoking Refusal Self-Efficacy Scale, and the Smokers’ Identity Identification Scale. Data were analyzed using Pearson correlation and chain mediation analysis (PROCESS Macro Model 6) with 5,000 bootstrap resamples.

**Results:**

Physical activity was significantly correlated with lower nicotine dependence level. The indirect association between physical activity and nicotine dependence level through smoking refusal self-efficacy was statistically significant, as was the indirect association through smokers’ identity identification, and the sequential indirect association through both mediators.

**Conclusion:**

Physical activity is associated with lower nicotine dependence level among female college smokers, and this association operates through indirect pathways via smoking refusal self-efficacy and smoker’ identity identification. These findings provide preliminary correlational evidence for the psychological pathways linking physical activity to nicotine dependence level, though longitudinal research is needed to establish causality.

## 1 Introduction

Nicotine dependence is a chronic relapsing disorder characterized by persistent smoking, intense craving, withdrawal symptoms, and diminished control. It not only significantly impairs respiratory, cardiovascular, and immune functions but also exhibits high comorbidity with various diseases such as lung cancer, depression, and anxiety (Cosci et al., 2011). Female college smokers are at a critical juncture of physiological maturation and social role transition. Despite their high educational attainment, they face multiple stressors including academic pressure, interpersonal challenges, and employment uncertainty, leading to rising smoking rates and increased nicotine dependence level among female college smokers, which has become a public health challenge on campuses (Tan et al., 2020). Previous research suggests that physical activity may influence nicotine dependence through potential mechanisms such as modulating the mesolimbic dopamine pathway and alleviating withdrawal symptoms and negative emotions (Janse van Rensburg et al., 2009), although these physiological pathways were not directly tested in the present study. However, exercise type, intensity, and individual differences may moderate its effects, with certain forms potentially causing adverse impacts by reinforcing body consciousness (Gossin et al., 2020). Although previous studies have examined the associations between physical activity and smoking-related outcomes, and separately investigated the roles of self-efficacy and identity in smoking behavior, few have tested whether these two psychological constructs sequentially mediate the physical activity–nicotine dependence relationship among female college smokers. To address this gap, the present study aims to systematically examine how physical activity may be associated with nicotine dependence through the serial pathways of smoking refusal self-efficacy and smoker identity, providing theoretical and empirical foundations for tailored smoking cessation programs integrating exercise interventions, cognitive-behavioral training, and motivational interviewing.

## 2 Research hypothesis

### 2.1 Predictive effects of physical activity and nicotine dependence level among

#### 2.1.1 Female college smokers

Physical activity refers to bodily movements involving planned, structured, and repetitive physical actions aimed at improving or maintaining one or more components of physical health (American College of Sports Medicine, 2018). Firstly, existing studies indicate that acute moderate-intensity aerobic exercise may rapidly upregulate activity in the mesolimbic dopamine pathway, potentially reducing immediate cravings and withdrawal symptoms (Ni et al., 2022). Secondly, long-term regular physical activity has been associated with enhanced cardiorespiratory fitness and basal metabolic rate, which may accelerate the clearance of nicotine and its metabolites, potentially reducing perceived dependence levels (Cai et al., 2023). Moreover, physical activity has been linked to the secretion of *β*-endorphins and brain-derived neurotrophic factor (BDNF), which may alleviate negative emotions such as anxiety and depression, potentially weakening emotion-driven smoking motivation (Alizadeh Pahlavani, 2023). Finally, the exercise setting itself provides temporal occupation and alternative rewards; when training schedules, fitness socialization, or competition goals become core daily activities, the accessibility and priority of smoking behavior may be naturally diluted (Chen et al., 2022b). Additionally, improved exercise-related self-efficacy and body image may suppress cognitive conflicts between “continuing smoking” and “maintaining physical fitness” (Farris et al., 2016). These physiological mechanisms, however, were not measured in the present study and are discussed only as background literature. Based on this, Hypothesis H1 is proposed: physical activity exhibits a negative association with nicotine dependence level among female college smokers.

### 2.2 Prediction of the mediating effect of smoking refusal self-efficacy

Smoking refusal self-efficacy refers to smoker’ self-assessment of their confidence in resisting smoking when offered cigarettes or pressured to smoke by others (Langlois et al., 2006). The proposed mediation model is grounded in Bandura’s Social Cognitive Theory, which emphasizes the reciprocal interplay between personal beliefs, behavioral performance, and environmental influences. Within this framework, self-efficacy—confidence in one’s ability to execute a specific action—is conceptualized as a key mechanism through which behavioral interventions (such as physical activity) influence health outcomes. Existing studies suggest that regular aerobic exercise may enhance individuals’ confidence in self-control through improved cardiorespiratory function and emotional regulation capacity (Zhang et al., 2019). During exercise, the release of endorphins and dopamine may not only alleviate anxiety and depression during early smoking cessation but also foster the psychological reinforcement of “I can control my body,” thereby potentially strengthening the belief in saying “no” to social smoking pressures (Bruijnzeel, 2017). Furthermore, peer support and health-oriented environments within exercise settings may provide vicarious reinforcement, potentially enabling female smokers to integrate the “exerciser” identity with the “non-smoker” identity, further consolidating smoking refusal self-efficacy (Priebe et al., 2020). The physiological mechanisms mentioned above (e.g., endorphin and dopamine release) were not measured in the present study and are discussed only as background literature. Once enhanced, smoking refusal self-efficacy may associate with nicotine dependence level among female college smokers. High smoking refusal self-efficacy strengthens cognitive control over smoking urges (Ma et al., 2020). When exposed to typical situational cues (e.g., peer cigarette offering, exam stress), high-efficacy individuals are more likely to rapidly activate the self-suggestion of “I can refuse,” inhibiting immediate smoking impulses and consequently reducing daily cigarette consumption and shortening time to first cigarette upon waking (Evans et al., 2019). Simultaneously, females with high smoking refusal self-efficacy tend to choose alternatives such as exercise, deep breathing, or seeking social support to alleviate withdrawal symptoms rather than regulating emotions through smoking (Blevins et al., 2016). Based on this, Hypothesis H2 is proposed: smoking refusal self-efficacy exhibits a negative association with nicotine dependence level among female college smokers, thereby mediating the relationship between physical activity and nicotine dependence level among female college smokers.

### 2.3 Prediction of the mediating effect of smokers’ identity identification

Smoker’ identity identification refers to the extent to which individuals incorporate the smoker identity into their self-concept, encompassing their understanding of the meaning of the smoker identity and recognition of the actions required to be a smoker (Chen et al., 2023). At the physiological level, previous research has suggested that aerobic training may enhance cardiorespiratory fitness and basal metabolic rate, potentially causing “a healthy body” to replace “needing nicotine” as a self-referential frame (Guo, 2023). At the psychological level, peer support and shared goals in exercise settings may foster a “health-oriented community,” gradually integrating the “exerciser” role into the core self, which may marginalize smoking behavior as “not-self” action (Liu et al., 2019). At the behavioral level, fixed training schedules may occupy time and situational cues previously available for smoking, creating an “accessibility dilution” of cigarettes and further reducing the priority of smoking behavior in daily life scripts (Fang and Lin, 2003). These physiological pathways, however, were not measured in the present study and are discussed only as background literature. When smoker’ identity identification is weakened, it may associate with nicotine dependence level among female college smokers. Individuals with low identity identification may more readily perceive smoking cessation as “self-consistency” rather than “self-loss,” thereby mobilizing cognitive control resources to prioritize alternative strategies (e.g., exercise, deep breathing, social support) when facing peer cigarette offers or exam stress (Meijer et al., 2020). Conversely, those with high identity identification may tend to view smoking as a core expression of “who I am,” experiencing any quit attempt as identity threat and subsequently triggering compensatory smoking. Based on this, Hypothesis H3 is proposed: smokers’ identity identification exhibits a positive association with nicotine dependence level among female college smokers, thereby mediating the relationship between physical activity and nicotine dependence level among female college smokers.

Although both smoking refusal self-efficacy and smoker identity are conceptually related, they represent distinct constructs. Smoking refusal self-efficacy is a behavioral-level cognitive belief—confidence in executing a specific action (refusing cigarettes) in particular situations. Smoker identity, in contrast, is an identity-level self-concept—the degree to which the “smoker” label is integrated into one’s self-definition. This distinction between behavioral-level self-efficacy (drawn from Social Cognitive Theory) and identity-level self-concept (drawn from Identity Theory) provides the theoretical foundation for treating them as sequential mediators rather than interchangeable constructs. Self-efficacy addresses “Can I refuse smoking?” whereas identity addresses “Who am I?”—two fundamentally different psychological processes. Thus, although empirically correlated, they are theoretically separable and both included in the model.

### 2.4 Prediction of the chain-mediated effect of smoking refusal self-efficacy and smokers’ identity identification

**Figure 1:**
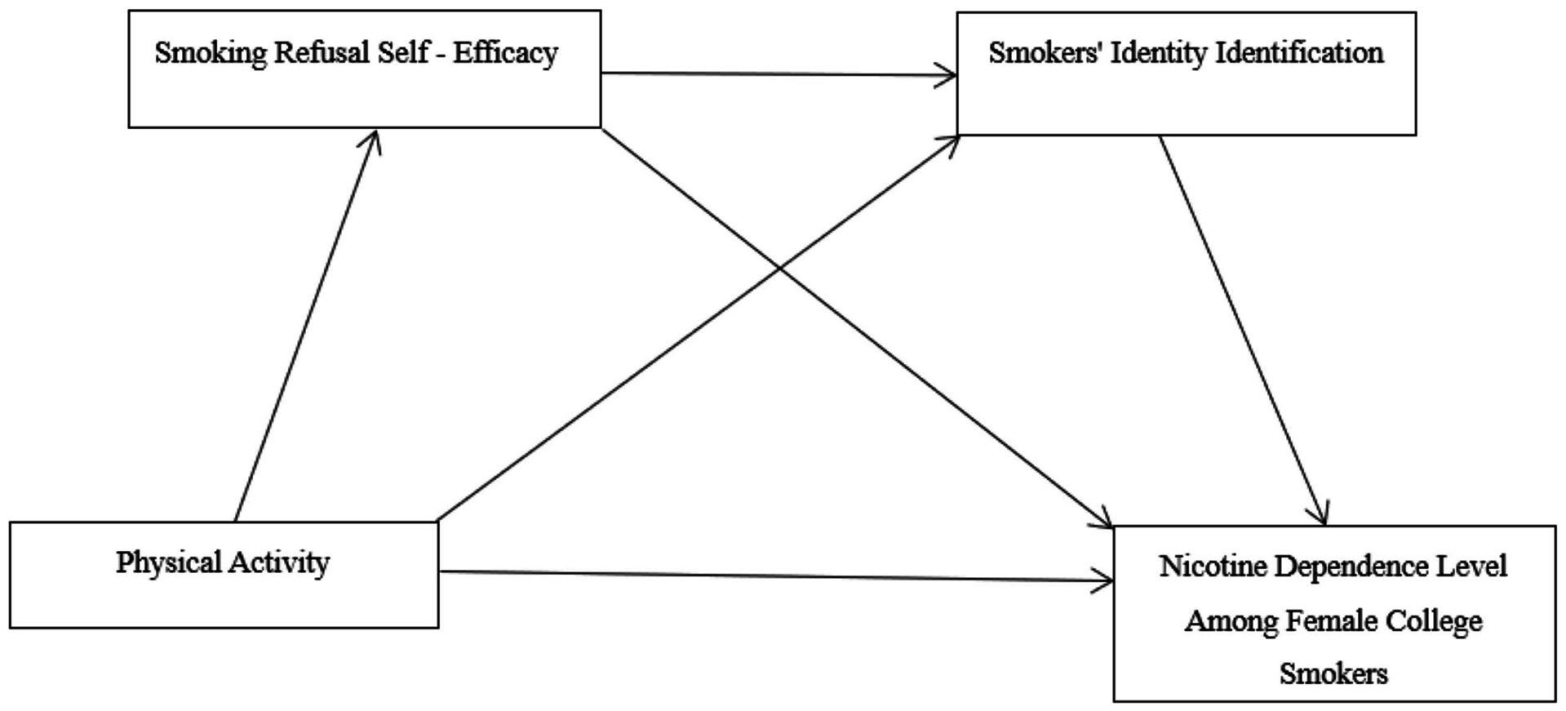
Diagram of the hypothetical model.

Smoking refusal self-efficacy continuously provides successful experiences of “I can refrain from smoking,” conflicting with the pre-existing self-schema of “I am a smoker.” This conflict prompts individuals to reclassify smoking behavior as an “occasional act” rather than “self-definition,” thereby undermining the stability of identity labeling (Zhang et al., 2023). Each successful smoking refusal generates pride and mastery experiences that may negatively predict emotional attachment to the smoker identity, causing the “smoker” role to slide from the “core self” toward a “peripheral role” (Tombor et al., 2013). Higher smoking refusal self-efficacy may increase individuals’ tendency to proactively avoid smoking contexts and choose alternative activities, with the “de-smoking” of behavioral sequences further diluting identity cues (Gwaltney et al., 2005). When refusal behaviors are perceived by peers as symbols of health and self-discipline, external feedback may reinforce the “non-smoker” role identity and weaken belongingness to original smoking groups through social comparison mechanisms, ultimately leading to comprehensive weakening of smokers’ identity identification (van den Putte et al., 2009). Based on this, Hypothesis H4 is proposed as an exploratory indirect association: smoking refusal self-efficacy and smokers’ identity identification are sequentially and indirectly associated with the relationship between physical activity and nicotine dependence level among female college smokers, consistent with the chain mediation model (see Figure 1). However, due to the cross-sectional design, this pathway should be interpreted as an exploratory indirect association rather than a confirmed causal sequence.

## 3 Methodology

### 3.1 Research subject

This study employed a cross-sectional survey design. A cross-sectional design was chosen for several reasons. First, the primary aim of this study was to explore whether the proposed serial indirect pathways are statistically consistent with the data, rather than to establish causal ordering. As recommended by methodological scholars, cross-sectional mediation analysis is appropriate for preliminary theory testing when interpreted with caution. Second, given the exploratory nature of the proposed sequential model—which has not been previously tested among female college smokers—a cross-sectional design provides a feasible and efficient approach to identify potential psychological pathways before investing in costly longitudinal or experimental follow-up studies. Third, practical constraints (e.g., participant recruitment, retention, and academic calendar) made a longitudinal design challenging for the current study. However, we fully acknowledge that cross-sectional data cannot establish temporal precedence or causal order. Therefore, all mediation results are interpreted as exploratory indirect associations, and this limitation is explicitly addressed in the Discussion section.

Participants were female college students recruited from 30 universities across various regions of China using a stratified random sampling strategy. Stratification was based on two criteria: (1) geographic region (universities were selected from eastern, central, and western China); and (2) university type (comprehensive, normal, and science/engineering universities). Within each stratum, universities were randomly selected, resulting in a total of 30 universities.

Current smokers were defined as individuals who reported smoking at least one cigarette per day in the past 30 days. Inclusion criteria were: (1) female college students currently enrolled in a participating university; (2) aged 18–25 years; (3) met the smoking definition above. Exclusion criteria were: (1) former smokers who had quit for more than 6 months; (2) individuals with a history of psychiatric disorders or current use of smoking cessation medications.

Recruitment was conducted through online platforms. Research assistants contacted student affairs offices at each selected university and distributed the questionnaire link via class WeChat groups and university online forums. The questionnaire was administered using an online survey platform (Wenjuanxing). The survey was anonymous, voluntary, and no compensation was provided. All participants provided electronic informed consent before starting the questionnaire.

**Figure 2:**
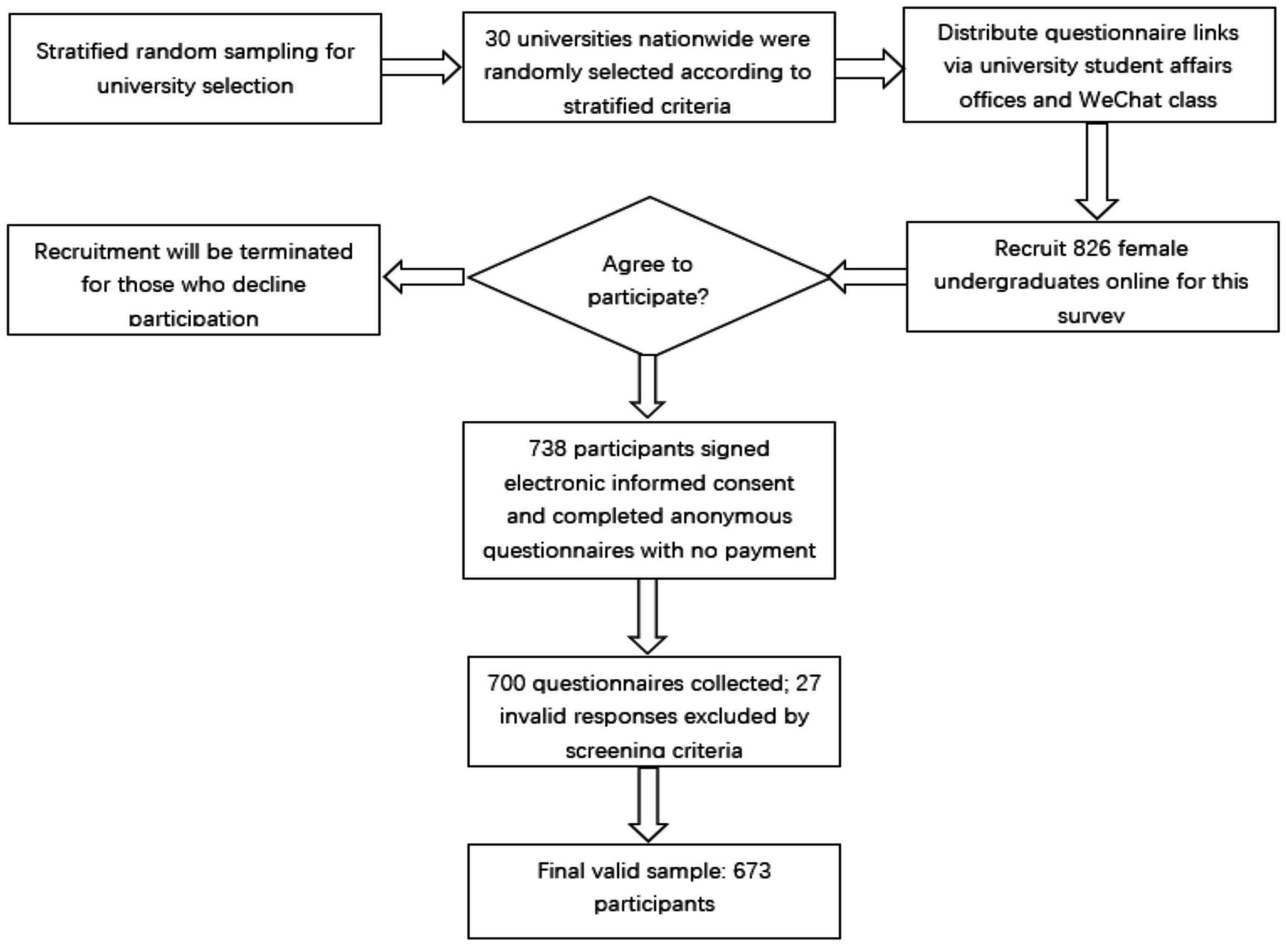
Sampling flow diagram.

The response process was as follows: of 826 students initially approached through the online channels, 738 agreed to participate and accessed the questionnaire link (initial consent rate: 89.3%); 700 completed questionnaires were submitted; 27 were excluded based on the following quantitative criteria: (1) incompleteness—questionnaires with more than 20% missing items (i.e., ≥2 missing items on scales with 6–9 items) were excluded (*n* = 21); (2) extreme response patterns—questionnaires with uniform responses across an entire scale (e.g., all items endorsed as “3” on a 5-point scale) were excluded (*n* = 4); (3) insufficient completion time—questionnaires completed in less than 60 s were excluded (*n* = 2), yielding 673 valid responses (valid response rate: 91.1% of submitted; 81.5% of consented). A simplified sampling flow diagram is provided as Figure 2.

To assess whether exclusions introduced systematic bias, we compared excluded (*n* = 27) and included (*n* = 673) participants on key demographic variables (age, grade, and smoking history). Independent-samples *t*-tests and chi-square tests revealed no significant differences between the two groups on any of these variables (all *p* > 0.05), indicating that the exclusions did not substantively affect sample representativeness.

### 3.2 Research method

#### 3.2.1 Questionnaire survey method

This study employed a cross-sectional survey design using a questionnaire survey method. The questionnaire was administered online via the Wenjuanxing platform. The survey was anonymous and voluntary; all participants provided electronic informed consent before starting. The questionnaire took approximately 15–20 min to complete. Study setting, participants, and recruitment procedures are described in Section 2.1. The study was approved by the Ethics Committee of Liaoning University of International Business and Economics.

#### 3.2.2 Mathematical and statistical method

Data were analyzed using SPSS 29.0. Preliminary analyses included: (1) reliability assessment using Cronbach‘s alpha coefficients for each scale; (2) common method bias assessment using Harman’s single-factor test; (3) descriptive statistics for demographic characteristics; and (4) Pearson correlation analysis among all study variables. To test the hypothesized chain mediation model, PROCESS Macro Model 6 was employed with 5,000 bootstrap resamples to estimate indirect effects and their 95% bias-corrected confidence intervals. Academic year was included as a control variable based on theoretical relevance (see Section 3.4). Prior to the main analyses, we examined the assumptions of multiple regression. Normality of residuals was assessed using the Shapiro–Wilk test and visual inspection of *Q*–*Q* plots; homoscedasticity was checked using scatterplots of standardized residuals against predicted values; linearity was examined through partial regression plots; influential cases were identified using Cook’s distance (values < 1 considered non-influential); and residual independence was verified using the Durbin–Watson statistic (values between 1.5 and 2.5 considered acceptable). These diagnostic checks revealed no substantial violations of regression assumptions. Multicollinearity was further assessed using VIF and tolerance values, which are reported in the Results section (see Section 3.5).

### 3.3 Research instruments

#### 3.3.1 Physical Activity Level Scale

The Physical Activity Level Scale, originally developed by Hashimoto Kimio (Liang, 1994), was used to assess participants’ physical activity during the previous week. The scale evaluates three dimensions: intensity (5-point: 1 = light to 5 = heavy), duration (5-point: 1 = ≤10 min to 5 = ≥60 min), and frequency (5-point: 1 = <1 time/week to 5 = ≥4 times/week). Total scores were calculated using the formula: Intensity × Frequency × Duration − 1, yielding a possible range of 0 to 124. Higher scores indicate higher levels of physical activity. The Chinese version of this scale has demonstrated acceptable validity and reliability in previous research among Chinese college students (Han et al., 2024). In the present sample, the scale showed good internal consistency (Cronbach’s *α* = 0.85).

#### 3.3.2 Nicotine Dependence Level Scale

The Fagerström Test for Nicotine Dependence (FTND), as revised by Pan et al. (2010), was employed. This scale comprises 6 items assessing cigarettes per day, time to first cigarette after waking, difficulty refraining from smoking in prohibited places, smoking when ill, and other dependence-related behaviors. Total scores were calculated by summing all item scores (possible range: 0–10), with higher scores indicating greater nicotine dependence. Following standard guidelines, dependence levels were categorized as: low dependence (0–3), moderate dependence (4–6), and high dependence (7–10). The Chinese version of the FTND has demonstrated acceptable reliability and validity in previous research (Pan et al., 2010). In the present sample, the scale showed acceptable internal consistency (Cronbach’s *α* = 0.72).

#### 3.3.3 Smoking Refusal Self-Efficacy Scale

The Smoking Refusal Self-Efficacy Scale developed by Langlois et al. (2006). This scale consists of 9 items rated on a 4-point Likert scale, requiring participants to indicate to what extent they could resist smoking in specific situations. Higher scores reflect greater.

Smoking Refusal Self-Efficacy. In this study, the scale demonstrated a Cronbach’s *α* coefficient of 0.95.

#### 3.3.4 Smokers’ Identity Identification Scale

The Smokers’ Identity Identification Scale developed by Moan and Rise was employed (Moan and Rise, 2005). This scale comprises 4 items rated on a 7-point Likert scale, with higher scores indicating stronger Smokers’ Identity Identification. In this study, the scale yielded a Cronbach’s *α* coefficient of 0.88.

## 4 Research results

### 4.1 Control and test of common method bias

To address potential common method bias (CMB), several procedural remedies were implemented during the research design: (1) all questionnaires were administered anonymously; (2) some scales contained reverse-scored items; (3) the survey instructions emphasized that there were no right or wrong answers to reduce social desirability pressures. Statistically, Harman‘s single-factor test was conducted by entering all scale items into an exploratory factor analysis without rotation. The results showed six factors with eigenvalues greater than 1, and the largest factor explained 33.8% of the total variance, below the 40% threshold. However, we acknowledge that Harman’s test alone is insufficient to fully rule out CMB. Thus, we interpret our findings conservatively, and common method bias is explicitly addressed as a limitation in the Discussion section.

### 4.2 Sample size justification

To justify the sample size, we conducted an *a priori* power analysis using GPower 3.1.9.2 for linear multiple regression (fixed model, *R^2^* deviation from zero). Based on a conservative medium effect size (*f^2^* = 0.15), significance level *α* = 0.05, desired power (1 − *β*) = 0.80, and number of predictors *k* = 3, the minimum required sample size was 77. Using a smaller expected effect size (*f^2^* = 0.02, small effect), the required sample size was 550 for power = 0.80. Our final sample (*N* = 673) exceeds both estimates, indicating adequate statistical power to detect small to medium effects in the mediation model.

### 4.3 Descriptive statistics

Table 1 provides a demographic analysis of 673 subjects, breaking down data across three dimensions: household smoking status, smoking history, and grade. In terms of household smoking status, 550 households (81.7%) have smokers, while 123 households (18.3%) have no smokers. Regarding smoking history, the largest group consists of those with a 1-year smoking history, totaling 306 participants (45.5%), followed by 130 with 3 years (19.3%), 125 with 2 years (18.6%), and 112 with 4 years (16.6%). For grade distribution, there are 178 freshmen (26.4%), 167 sophomores (24.8%), 167 juniors (24.8%), 139 seniors (20.7%), and 22 graduate students (3.3%). This table clearly presents the demographic distribution characteristics of the sample, providing basic data support for subsequent research, as shown in the table.

**Table 1:** Demographic analysis.

Attribute	Category	Quantity	Percentage
Family smoking status	Household with smokers	550	81.7%
	Household without smokers	123	18.3%
Smoking history	1 year	306	45.5%
	2 years	125	18.6%
	3 years	130	19.3%
	4 years	112	16.6%
Grade	Freshman	178	26.4%
	Sophomore	167	24.8%
	Junior	167	24.8%
	Senior	139	20.7%
	Graduate students	22	3.3%

Table presents demographic characteristics of 673 participants, including household smoking status (with/without smokers), smoking history (1–4 years), and grade distribution. Data are shown as counts (*n*) and percentages (%), reflecting the distribution of key variables.

Additionally, independent samples *t*-tests were conducted on family smoking status to examine significant differences in all variables across different family smoking status groups. Statistical results are presented in Table 2.

**Table 2:** Independent samples *t*-test.

Attribute	Category	*N*	*M ± SD*	*t*	*p*
Physical activity	Household with smokers	550	15.136 ± 14.276	−0.819	*p* = 0.413
	Household without smokers	123	16.317 ± 15.223		
Dependence level among female college smokers	Household with smokers	550	2.020 ± 0.382	1.490	*p* = 0.137
	Household without smokers	123	1.963 ± 0.387		
Smoking refusal self-efficacy	Household with smokers	550	2.117 ± 1.067	−0.361	*p* = 0.718
	Household without smokers	123	2.155 ± 1.094		
Smokers’ identity identification	Household with smokers	550	3.862 ± 1.127	1.021	*p* = 0.308
	Household without smokers	123	3.748 ± 1.079		

*N* denotes sample size, *M* ± *SD* represents mean ± standard deviation. *T* is the *t*-statistic and *p* is the *p*-value.

Results from the independent samples *t*-tests indicated no statistically significant differences in all core variables across family smoking status groups (all *p* > 0.05). Despite this, we initially retained family smoking status as a control variable in the mediation model to examine whether it influenced the estimated indirect effects. The results showed that inclusion of family smoking status did not change the direction or significance of any pathway coefficients. Given that (1) the inclusion of this variable did not affect the model estimates, (2) the theoretical focus of the present study is on psychological mechanisms (self-efficacy and identity) rather than distal environmental factors, and (3) the study sample is drawn from a university setting where peer and campus environmental influences may supersede family background, we excluded it from the final model to maintain parsimony. This decision was based on theoretical and statistical considerations, not solely on the non-significant *t*-test results.

We acknowledge that other potential confounding variables—such as socioeconomic status, alcohol use, stress, depressive symptoms, peer smoking, body mass index, exercise participation motives, and psychiatric history—were not measured in this study and thus could not be included as covariates. These variables are known to influence nicotine dependence and their omission may introduce omitted-variable bias. However, given the exploratory nature of this cross-sectional study and the specific focus on psychological pathways (self-efficacy and identity), we prioritized theoretical parsimony and the primary research question over exhaustive covariate adjustment. We have explicitly acknowledged this limitation in the Discussion section, and recommend that future longitudinal studies incorporate a broader range of potential confounders to strengthen causal inferences.

### 4.4 ANOVA single factor analysis

One-way ANOVA was initially conducted to examine differences in physical activity, nicotine dependence level among female college smokers, smoking refusal self-efficacy, and smokers’ identity identification across academic years. Results revealed significant differences in smoking refusal self-efficacy and smokers’ identity identification among female college smokers of different academic years (both *p* < 0.05), as detailed in Table 3.

**Table 3:** One-way analysis of ANOVA of different grades.

Relevant variable	Grade	*M ± SD*	*F*	*p*
Physical activity	1 (178)	13.416 ± 12.979	2.315	*p* = 0.056
	2 (167)	15.659 ± 13.780		
	3 (167)	17.246 ± 17.013		
	4 (139)	16.000 ± 14.083		
	5 (22)	10.227 ± 8.783		
Dependence level among female college smokers	1 (178)	2.042 ± 0.381	2.154	*p* = 0.073
	2 (167)	2.015 ± 0.387		
	3 (167)	2.012 ± 0.387		
	4 (139)	1.941 ± 0.364		
	5 (22)	2.129 ± 0.418		
Smoking refusal self-efficacy	1 (178)	1.635 ± 0.365	3.301	*p<*0.01
	2 (167)	1.641 ± 0.354		
	3 (167)	1.623 ± 0.318		
	4 (139)	1.556 ± 0.358		
	5 (22)	1.692 ± 0.573		
Smokers’ identity identification	1 (178)	4.348 ± 0.476	8.200	*p<*0.01
	2 (167)	4.295 ± 0.482		
	3 (167)	4.335 ± 0.461		
	4 (139)	4.265 ± 0.472		
	5 (22)	4.424 ± 0.439		

*M* ± *SD* indicates mean ± standard deviation. *F* is the *F*-statistic for testing the null hypothesis of equal group means. *p* is the *p*-value; *p* < 0.05 suggests statistically significant differences among groups.

One-way ANOVA results indicated that both smoking refusal self-efficacy and smokers’ identity identification exhibited statistically significant differences across academic years (both *p* < 0.01). To clarify specific inter-year variations, *post-hoc* multiple comparisons were conducted to precisely delineate differences in these two variables among academic years. Consequently, academic year was incorporated as a control variable in subsequent regression analyses for smoking refusal self-efficacy and smokers’ identity identification. This approach provides robust statistical support for elucidating developmental trajectories of smoking-related psychological behaviors among female college smokers across academic years. Results of *post-hoc* multiple comparisons are presented in Tables 4, 5.

**Table 4:** Multiple comparisons of smoking refusal self-efficacy among female college smokers across academic years.

Grade	1 (1.635)	2 (1.641)	3 (1.623)	4 (4.001)
5 (1.692)	−0.05709	−0.05120	−0.06916	0.13609
4 (1.556)	0.07903	0.08492	0.06696	
3 (1.623)	0.01208	0.01796		
2 (1.641)	−0.00589			

The significance level for mean differences was set at 0.05.

**Table 5:** Multiple comparisons of smokers’ identity identification among female college smokers across academic years.

Grade	1 (4.348)	2 (4.295)	3 (4.335)	4 (4.265)
5 (4.424)	−0.07593	−0.12883	−0.08891	0.15909
4 (4.265)	0.08308	0.03018	0.06990	
3 (4.335)	0.01299	−0.03992		
2 (4.295)	0.05291			

The significance level for mean differences was set at 0.05.

Subsequently, one-way ANOVA was performed to examine differences in physical activity, nicotine dependence level among female college smokers, smoking refusal self-efficacy, and smokers’ identity identification across groups with different smoking histories. Results indicated no statistically significant differences in any variables among female college smokers from different smoking history groups (all *p* > 0.05). The data are presented in Table 6.

**Table 6:** One-way analysis of ANOVA of smoking history.

Relevant variable	Smoking history	*M ± SD*	*F*	*p*
Physical activity	1 (306)	15.033 ± 14.231	1.527	*p* = 0.206
	2 (125)	17.776 ± 15.826		
	3 (130)	14.731 ± 13.741		
	4 (112)	14.241 ± 14.128		
Dependence level among female college smokers	1 (306)	2.009 ± 0.381	0.984	*p* = 0.400
	2 (125)	2.059 ± 0.391		
	3 (130)	1.986 ± 0.379		
	4 (112)	1.987 ± 0.384		
Smoking refusal self-efficacy	1 (306)	2.141 ± 1.056	0.348	*p* = 0.791
	2 (125)	2.041 ± 1.046		
	3 (130)	2.123 ± 1.074		
	4 (112)	2.172 ± 1.145		
Smokers’ identity identification	1 (306)	3.780 ± 1.142	0.773	*p =* 0.510
	2 (125)	3.952 ± 1.053		
	3 (130)	3.882 ± 1.096		
	4 (112)	3.836 ± 1.150		

*M* ± *SD* indicates mean ± standard deviation. *F* is the *F*-statistic for testing the null hypothesis of equal group means. *p* is the *p*-value; *p* < 0.05 suggests statistically significant differences among groups.

One-way ANOVA results revealed that for all variables examined across different smoking history groups, the corresponding *p*-values exceeded 0.05, indicating that smoking history exerted no statistically significant influence on any variables and no significant differences were observed. Consequently, *post-hoc* multiple comparisons were deemed unnecessary. The current findings sufficiently demonstrate consistent characteristics of smoking-related psychological behaviors among female college students across the dimension of smoking history. Similarly, to prevent potential interference from redundant variables and to streamline the analytical model, smoking history was not incorporated as a control variable in subsequent analyses of this study (see Table 7).

**Table 7:** Pearson correlation analysis.

Attribute	Physical activity	Nicotine dependence level among female college smokers	Smoking refusal self-efficacy	Smokers’ identity identification
Physical activity	1			
Nicotine dependence level among female college smokers	−0.280**	1		
Smoking refusal self-efficacy	0.127**	−0.260**	1	
Smokers’ identity identification	−0.129**	0.248**	−0.912**	1

Significant correlation at the 0.01 level (two-tailed).

### 4.5 Correlation analysis between variables

Correlation analysis was performed using SPSS software by incorporating four variables—physical activity, nicotine dependence level among female college smokers, smoking refusal self-efficacy, and smokers’ identity identification—into the dataset. Calculation of Pearson correlation coefficients revealed a significant negative correlation between physical activity and nicotine dependence level among female college smokers, indicating that higher frequency of physical activity was associated with lower levels of nicotine dependence. Furthermore, physical activity showed a significant positive correlation with smoking refusal self-efficacy, while smoking refusal self-efficacy was significantly negatively correlated with nicotine dependence level among female college smokers, suggesting that individuals engaging in regular physical activity tend to possess higher smoking refusal self-efficacy, which in turn is correlated with lower nicotine dependence. Additionally, physical activity demonstrated a significant negative correlation with smokers’ identity identification, whereas smokers’ identity identification was significantly positively correlated with nicotine dependence level among female college smokers, indicating that regular physical activity participation is associated with weaker smoker identity, which in turn is associated with higher nicotine dependence. Finally, a significant negative correlation was observed between smoking refusal self-efficacy and smokers’ identity identification, implying that higher smoking refusal self-efficacy corresponds to weaker smokers’ identity identification. These results suggest that physical activity, smoking refusal self-efficacy, and smokers’ identity identification are associated with nicotine dependence level among female college smokers, though causal inferences cannot be drawn due to the cross-sectional design.

As shown in Table 8, the chain mediating effects were tested using Model 6 from Hayes’ SPSS PROCESS macro. Based on theoretical considerations, academic year was included as a control variable in the equations for smoking refusal self-efficacy (M1) and smoker identity (M2), because grade level reflects developmental differences in self-cognition and social role identification that may influence these psychological constructs (Rosa and Aloise-Young, 2015). However, academic year was not included in the equation for nicotine dependence (Y) for two theoretical reasons: (1) nicotine dependence is primarily a physiological and behavioral outcome, and any effects of academic year on dependence are hypothesized to operate through the two psychological mediators rather than directly; (2) controlling for grade in the Y equation would risk overcontrolling, as it would partial out variance already explained by M1 and M2, potentially masking the indirect effects of interest. This covariate strategy was determined *a priori* based on theoretical reasoning rather than on the significance or non-significance of preliminary statistical tests.

**Table 8:** Regression analysis between variables.

Regression equation		Overall fit index			Significance of regression coefficients		
Outcome variable	Predictor variable	*R*	*R^2^*	*F*	*β*	*t*	*p*
Smoking refusal self-efficacy (*M1*)	Physical activity (*X*)	0.591	0.349	180.127	0.095	3.028	<0.01
	Grade				0.579	1.102	0.27
Smokers’ identity identification (*M2*)	Physical activity (*X*)	0.915	0.837	1120.418	−0.018	−3.426	<0.01
	Smoking refusal self-efficacy *(M1)*				−0.889	−44.973	<0.001
	Grade				−0.035	−1.789	0.074
Nicotine dependence level among female college smokers *(Y)*	Physical activity *(X)*	0.582	0.339	44.827	−0.245	−6.771	<0.001
	Smoking refusal self-efficacy*(M1)*				−0.258	−2.863	<0.01
	Smokers’ identity identification *(M2)*				0.060	2.647	<0.05

“Overall Fit Index” includes R (multiple correlation), *R*^2^ (coefficient of determination), *F* (model significance test). “Significance of Regression Coefficients” includes *β* (standardized regression coefficient), *t* (individual predictor test), and *p* (significance level; *p* < 0.05 indicates statistical significance). All reported *β* values are standardized coefficients.

The results demonstrated that: physical activity was significantly positively associated with smoking refusal self-efficacy (*β* = 0.095, *p* < 0.01); physical activity was significantly negatively associated with smokers’ identity identification (*β* = −0.018, *p* < 0.01); smoking refusal self-efficacy was significantly negatively associated with nicotine dependence level among female college smokers (*β* = −0.258, *p* < 0.01); smokers’ identity identification was significantly positively associated with nicotine dependence level among female college smokers (*β* = 0.060, *p* < 0.05); smoking refusal self-efficacy was significantly negatively associated with smokers’ identity identification (*β* = −0.889, *p* < 0.001); and physical activity was significantly negatively associated with nicotine dependence level among female college smokers (*β* = −0.245, *p* < 0.001).

To assess multicollinearity between the two mediators, we calculated variance inflation factors (VIF) and tolerance values. The VIF was 10.82 and tolerance was 0.092, exceeding the conventional threshold of VIF > 10, indicating severe multicollinearity between smoking refusal self-efficacy and smoker identity. This is consistent with the extremely high correlation observed (*r* = −0.912). Accordingly, the chain mediation results involving these two constructs should be interpreted with caution, and this issue is explicitly addressed as a limitation in the Discussion section.

### 4.6 Chain-mediated effects of smoking refusal self-efficacy and smokers’ identity identification between physical activity and nicotine dependence level among female college smokers

**Figure 3:**
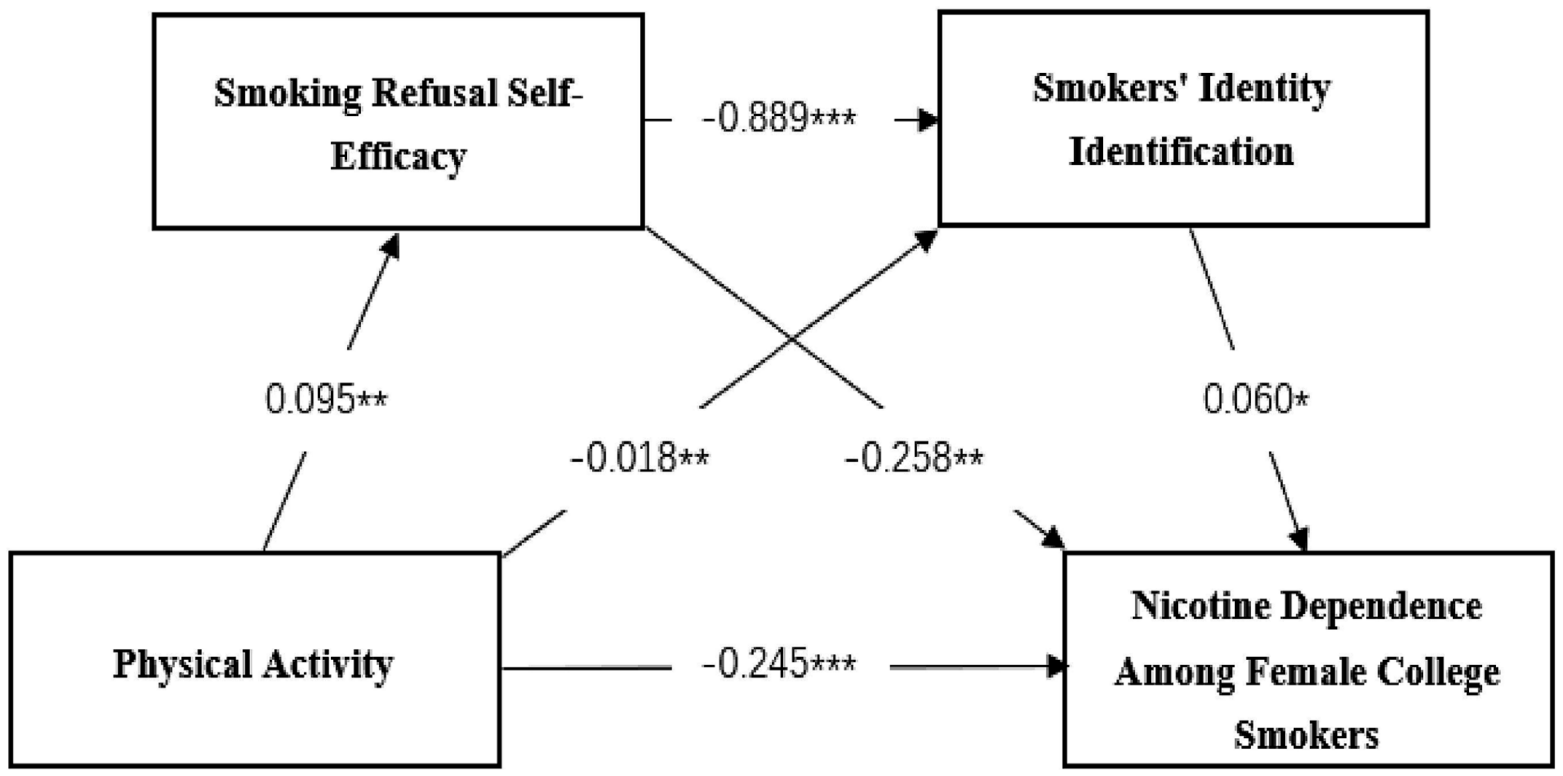
Serial mediation model with standardized path coefficients. Path coefficients are standardized regression coefficients (*β*), with significance levels indicated as follows: ***p < 0.001, **p < 0.01, *p < 0.05. All coefficients are derived from the updated regression analyses in Table 8. *X* = Physical Activity; *M1* = Smoking Refusal Self-Efficacy; *M2* = Smokers’ Identity Identification; *Y* = Nicotine Dependence Level among Female College Smokers. Academic year was included as a covariate in the *M1* and *M2* equations.

Smoking refusal self-efficacy and smokers’ identity identification played a chain mediating role between physical activity and nicotine dependence level among female college smokers. Physical activity reduced nicotine dependence level among female college smokers by enhancing smoking refusal self-efficacy, which subsequently weakened smokers’ identity identification. This chain mediating effect indicates that physical activity not only directly exerts a negative influence on nicotine dependence level among female college smokers, but also indirectly affects it through psychological mechanisms (smoking refusal self-efficacy and smokers’ identity identification). It should be noted, however, that the indirect effects accounted for only 12.16% of the total effect, indicating that the majority of the association remains unexplained by the two mediators. The statistical results are presented in Table 9, and the conceptual model is shown in Figure 3.

**Table 9:** Chain mediation effects table.

Effect (scientific phenomenon)	Trails	Efficiency value	Standard error	*LLCI*	*ULCI*	Efficiency ratio
Aggregate effect		−0.0074	0.0010	−0.0093	−0.0054	100%
Direct effect	Direct path	−0.0065	0.0010	−0.0084	−0.0046	87.84%
Total indirect effect		−0.0009	0.0003	−0.0015	−0.0003	12.16%
Indirect effect	Path 1	−0.0004	0.0002	−0.0012	−0.0001	5.41%
	Path 2	−0.0002	0.0001	−0.0010	−0.0002	2.70%
	Path 3	−0.0003	0.0002	−0.0015	−0.0006	4.05%

Table shows chain mediation effects. Columns: effect types, paths, effect size, variability, significance (via LLCI/ULCI), and ratio of total effect.

## 5 Discussion

### 5.1 Group difference analyses

The absence of significant differences across family smoking status groups may be explained by several factors, though these interpretations remain speculative. Psychologically, it is possible that regular physical activity strengthens individuals’ “healthy self-identity” (Zhang Y. et al., 2024), which may dilute the implicit influence of family smoking environments (Granero-Jiménez et al., 2022). The psychological resilience accumulated through exercise may also enable individuals to view family smoking behavior as personal choices of others rather than as determinants of their own behavior (Yang et al., 2024). In addition, female college students are in a stage of developing independence, where the influence of social circles and campus culture may surpass that of family. Exercise-formed social circles typically emphasize health (Roberts et al., 2012), which may further diminish the role of family smoking status. It is also possible that the null findings reflect measurement limitations (e.g., single-item assessment of family smoking status) or the relatively low prevalence of non-smoking families in the sample (18.3%), which may have reduced statistical power to detect group differences. Physiologically, while previous research has suggested that improved cardiorespiratory function and endorphin release may play a role (Wang and Hu, 2023), these mechanisms were not measured in the present study and remain speculative. Alternative explanations, such as unmeasured confounding variables (e.g., peer influence, campus environment, personal health beliefs), should also be considered. Future research with more comprehensive measures of family and social environmental factors is needed to clarify these null findings.

The significant differences in smoking refusal self-efficacy and smokers’ identity identification across academic years may reflect developmental differences in self-cognition and social role identification among college students. Lower-year students, having just left the highly disciplined high school environment, may rely more heavily on external social cues in forming their smoker identity—facing smoking behaviors of roommates or club members, they easily default to smoking behaviors due to psychological needs for “group integration,” forming vague and changeable identity identification (Rosa and Aloise-Young, 2015). Meanwhile, their understanding of smoking hazards remains superficial, lacking firm self-persuasion basis when encountering cigarette offering or group smoking scenes, resulting in weaker smoking refusal self-efficacy (Jiang et al., 2015). In contrast, upper-year students enter a self-integration period. With clearer professional directions and career planning, they actively incorporate smoking behavior into a “self-image management” framework. They consider whether smoking conflicts with long-term goals like “professional image” or “health reserve.” If judged “incompatible,” they strengthen rejection of smoking behavior, significantly enhancing self-confidence in refusal (smoking refusal self-efficacy) (Tate et al., 2021). Even if smoking behavior persists, they develop more solidified cognition based on long-term experience (e.g., “smoking is my way of relieving stress”), making smokers’ identity identification markedly different from the vague state of lower years. Additionally, upper-year students’ social circles focus more on professional fields or future development partners, with group attitudes toward smoking becoming more diversified (Lakon and Valente, 2012), reducing “follow-the-crowd” pressure and further amplifying differences in self-cognition and behavioral control compared to lower years.

These interpretations, however, are speculative and alternative explanations cannot be ruled out. For example, the null findings may reflect the relatively short smoking history among this sample (majority ≤3 years), which may not be sufficient to produce measurable differences in psychological constructs (Greaves and Hemsing, 2009). Additionally, smoking among female college students may be more situational (e.g., gatherings, stress relief) rather than reflecting stable physiological dependence (Zhang, 2023), which may weaken the impact of smoking history on dependence-related psychological variables (Chen et al., 2024). Future longitudinal research is needed to examine whether smoking history exerts cumulative effects on these variables over longer time periods (Chen et al., 2023).

### 5.2 Main effect analysis of physical activity on nicotine dependence level among female college smokers

Physical activity was significantly associated with lower nicotine dependence level among female college smokers. The potential mechanisms may be considered through three dimensions: psychological regulation, behavioral substitution, and socio-ecological influences.

From the psychological perspective, regular exercise may reshape emotional regulation patterns and provide alternative coping strategies. Female college students may use exercise to alleviate anxiety, potentially substituting for smoking behavior as a means of emotion regulation. While previous research has suggested physiological mechanisms such as endorphin release and neurobiological changes (Santos et al., 2021), these pathways were not measured in the present study. Our findings are limited to the psychological constructs examined (smoking refusal self-efficacy and smoker identity) and do not provide evidence regarding neurobiological mechanisms. At the level of behavioral substitution and cognitive restructuring, exercise occupies the “behavioral space” for smoking (e.g., morning runs or gym sessions replacing smoking scenarios), weakening the psychological demand for smoking (van Rensburg et al., 2009). Simultaneously, it strengthens the “health practitioner” identity, leveraging “self-consistency” to reject the “smoker” role, thereby dismantling the behavioral and psychological foundations of nicotine dependence. From the socio-ecological system analysis, exercise guides individuals into “health-oriented” social circles (e.g., running groups, fitness communities). Group norms (opposing smoking, advocating health) create environmental pressure, prompting individuals to reduce smoking (Tie et al., 2023). The “health ecology” formed by campus sports facilities and club activities also strips away the social support soil for nicotine dependence, reducing dependence levels (Lisha and Sussman, 2010).

In summary, the negative effect of physical activity on nicotine dependence is essentially an “ecological substitution” of health behaviors for addictive behaviors. Through the synergy of neurophysiological regulation, psychological cognitive restructuring, and socio-ecological intervention, it systematically dismantles the foundation of nicotine dependence at the levels of neural mechanisms, behavioral patterns, identity identification, and social environment, profoundly reflecting the dynamic interaction value of the “physiological-psychological-social” system in individual health management.

### 5.3 Chain-mediated effect analysis of smoking refusal self-efficacy and smokers’ identity identification

The association between physical activity and nicotine dependence level among female college smokers was found to be statistically consistent with a chain mediation pathway of “physical activity → smoking refusal self-efficacy → smokers’ identity identification → nicotine dependence.” This pathway is consistent with a theoretical feedback loop of “behavioral input → psychological transformation → behavioral output”—with physical activity as the behavioral initiation point, potentially activating the individual’s psychological motivation for smoking refusal (smoking refusal self-efficacy), which may then relate to identity restructuring, and ultimately correlate with nicotine dependence behavior. This process suggests a possible transmission from external behavioral intervention to internal psychological restructuring.

Physical activity may serve as a catalyst for smoking refusal self-efficacy through “behavioral empowerment” and “cognitive association”: on one hand, the “self-control competence” developed through regular exercise (e.g., overcoming inertia to maintain exercise habits) may transfer to smoking behavior management, enabling female college students to develop the belief that “I am capable of refusing smoking” (Zhang Z. et al., 2024); on the other hand, exercise may strengthen the connection between “health behaviors” and “self-value,” transforming smoking refusal from a passive constraint to an active choice, thereby potentially enhancing smoking refusal self-efficacy (Wierts et al., 2023). As smoking refusal self-efficacy becomes progressively strengthened, the conflict between the “smoker” identity and the “health behavior practitioner” identity may become more pronounced. Driven by the need for “self-cognitive consistency,” female college students may actively deconstruct the “smoker” label—transitioning from passive acceptance of the “smoker” identity toward identification as “health-conscious individuals capable of refusing smoking,” ultimately achieving weakening of smokers’ identity identification. In essence, this process may represent a psychological adjustment made to resolve internal cognitive dissonance.

The core value of this chain mediation mechanism lies in its potential “dual inhibition” of nicotine dependence. At the behavioral level, enhanced smoking refusal self-efficacy may increase actual smoking refusal behaviors (e.g., proactively declining cigarettes in social situations), while reduced smokers’ identity identification may diminish “self-justification of smoking behavior” (Chen et al., 2022a). These combined effects may reduce the behavioral frequency of nicotine dependence. At the psychological level, their synergistic action may reconstruct the psychological meaning of smoking behavior—transforming “smoking” from a “dependency-driven behavior” to a “rejected behavior,” while substituting the psychological need for nicotine with healthy alternatives such as “using exercise to relieve stress,” thereby potentially achieving psychological dependence cessation (Ruslan et al., 2023). This mechanism suggests a potential logic through which physical activity may be associated with nicotine dependence and provides a preliminary dual-path framework of “exercise empowerment + identity reconstruction,” highlighting the potential synergistic value of the “physiological-psychological-social” system in health behavior interventions.

It should be noted, however, that the indirect effects accounted for only 12.16% of the total effect (Path 1: 5.41%; Path 2: 2.70%; Path 3: 4.05%), with the direct effect comprising 87.84% of the total association. This indicates that the majority of the physical activity–nicotine dependence association remains unexplained by the two proposed mediators. Thus, while the indirect pathways were statistically significant, their practical magnitude is limited. These findings provide preliminary evidence consistent with the proposed psychological pathways, but should not be interpreted as confirmed mechanisms or as direct support for specific intervention prescriptions. Future research with longitudinal designs is needed to establish whether these indirect associations reflect genuine psychological processes.

### 5.4 Practical implications and recommendations

Social Level: Tentative Implications for a “Sports-Smoking Control” Collaborative Framework. Based on the observed indirect associations through which physical activity is linked to lower nicotine dependence via smoking refusal self-efficacy and smoker identity, it may be useful to consider integrating physical activity promotion and smoking control education into adolescent health policies. Primary health institutions could consider offering exercise guidance and group-based support for smoking refusal self-efficacy, drawing on the principle that exercise-induced “behavioral empowerment” may transfer to smoking refusal beliefs (National Health Commission of the People’s Republic of China, 2017). Simultaneously, scientific dissemination through authoritative media about the benefits of exercise for self-control could help reduce social perceptions that normalize smoking and weaken environmental triggers for smoker identity, potentially fostering a more supportive social ecology for health behaviors.

University Level: Tentative Implications for Campus-Based Interventions. As a core environment for identity development, universities may consider designing interventions through curricula and student organizations. Physical education courses could include modules that use team sports and endurance activities to cultivate “behavioral mastery experiences” (Zhang, 2013), combined with smoking refusal scenario simulations to enhance self-efficacy in social situations. Fitness clubs and smoking control associations might collaborate on activities that help students integrate the “sports participant” identity with a “non-smoker” identity. Such group interactions may strengthen self-cognitive consistency and facilitate the deconstruction of smoker identity, potentially targeting key psychological nodes in the pathway.

Family Level: Tentative Implications for Family Support. Family environments may indirectly influence these psychological processes by affecting individuals’ self-cognitive consistency. A support system based on shared physical activities and positive communication could be considered. Families might engage in joint exercise activities (e.g., walking, cycling, yoga) to reinforce the association between exercise and self-control through shared health behaviors (Rhodes et al., 2024). Parents could adopt nonjudgmental communication strategies, such as sharing personal experiences of overcoming unhealthy habits, rather than accusatory approaches (Rodrigues et al., 2018), which may reduce defensive reactions to the “smoker” label and create psychological safety for enhancing smoking refusal self-efficacy and identity reconstruction. These suggestions, however, are tentative and derived from the observed statistical associations; they should be tested in future intervention studies before implementation.

### 5.5 Limitations

Several limitations of this study should be acknowledged. First, the cross-sectional design prevents any causal or temporal inferences. Although we tested a serial mediation model, all variables were measured simultaneously, and the hypothesized temporal ordering (physical activity → self-efficacy → identity → dependence) remains assumed rather than empirically established. Reverse causation or unmeasured third-variable explanations cannot be ruled out.

Second, all data were collected via self-report questionnaires, which may introduce common method bias and social desirability bias. Although procedural remedies (anonymity, reverse-scored items, neutral instructions) and Harman‘s test were employed, we acknowledge that common method variance may still influence the observed associations.

Third, smoking status was defined by self-report without biochemical verification (e.g., exhaled carbon monoxide or urinary cotinine), which may introduce misclassification bias.

Fourth, the extremely high correlation between smoking refusal self-efficacy and smoker identity (*r* = −0.912) raises concerns about discriminant validity and multicollinearity. While we have reported VIF and tolerance diagnostics, this empirical overlap suggests that the two mediators may not function as fully distinct constructs in this sample, and the sequential distinction between them should be interpreted with caution.

Fifth, the sample was limited to female college smokers recruited from 30 universities across China. While this provides geographic diversity, the findings may not generalize to male smokers, non-college young adults, or smokers from other cultural contexts.

Sixth, physical activity was measured using a self-report scale rather than objective measures (e.g., accelerometers or fitness trackers), which may introduce recall bias and limit precision.

Seventh, several potentially important confounding variables—such as socioeconomic status, alcohol use, stress, depressive symptoms, peer smoking, body mass index, exercise participation motives, and psychiatric history—were not measured and thus could not be controlled for. Their omission may introduce omitted-variable bias.

Despite these limitations, the study provides preliminary correlational evidence for the proposed psychological pathways and identifies directions for future longitudinal and experimental research.

## 6 Conclusion

This study provides preliminary correlational evidence that physical activity is associated with lower nicotine dependence level among female college smokers. Beyond its direct association, the relationship was found to be sequentially and indirectly associated through the chain mediation of “smoking refusal self-efficacy → smokers’ identity identification”: this study provides preliminary correlational evidence that physical activity is associated with lower nicotine dependence among female college smokers, and this association was found to be sequentially and indirectly associated through smoking refusal self-efficacy and smokers’ identity identification. This pathway is consistent with the dynamic interaction of physiological-psychological-social systems and suggests tentative implications for smoking control interventions—society, universities, and families may consider strengthening support for physical activity, promoting smoking refusal self-efficacy training, and guiding identity reconstruction, thereby potentially assisting female college smokers in reducing nicotine dependence and fostering health behavior development. However, these findings should be interpreted with caution given the substantial empirical overlap between the two mediators. Longitudinal and experimental studies are needed to establish the temporal order and distinctiveness of these psychological pathways.

## Data Availability

The datasets presented in this study can be found in online repositories. The names of the repository/repositories and accession number(s) can be found at: https://pan.baidu.com/s/1I1rI0P-YLMmfDLu9hTvoKg.
